# Baseline Serum sLOX-1 Concentrations Are Associated with 2-Year Major Adverse Cardiovascular and Cerebrovascular Events in Patients after Percutaneous Coronary Intervention

**DOI:** 10.1155/2019/4925767

**Published:** 2019-10-20

**Authors:** Zi-wen Zhao, Yi-wei Xu, Shu-mei Li, Jin-jian Guo, Jian-min Sun, Ju-chang Hong, Liang-long Chen

**Affiliations:** ^1^Department of Cardiology, Union Hospital, Fujian Medical University, and Fujian Institute of Coronary Artery Disease, Fuzhou, China; ^2^Department of Cardiology, The 476 Clinical Department of Fuzhou General Hospital, China; ^3^Department of Cardiology, The Second People's Hospital of Fujian Province, Fujian University of Traditional Chinese Medicine, China

## Abstract

**Background:**

Soluble lectin-like oxidized low-density lipoprotein receptor-1 (sLOX-1) may be a potential biomarker of coronary artery disease (CAD) and stroke.

**Objective:**

We aimed to investigate the association and prognostic value of elevated sLOX-1 concentrations with regard to long-term major adverse cardiovascular and cerebrovascular events (MACCEs) in patients with CAD undergoing primary percutaneous coronary intervention (PCI).

**Methods:**

A total of 1011 patients were enrolled. Serum sLOX-1 concentrations were detected by the enzyme-linked immunosorbent assay (ELISA). Patients were followed for 2 years. Multivariate Cox regression and Kaplan-Meier survival curve were explored to assess the association between sLOX-1 and MACCEs. A receiver operating characteristic (ROC) curve was used to evaluate the diagnostic efficacy of sLOX-1.

**Results:**

Two-year MACCEs were associated with serum sLOX-1 concentrations (HR 1.278, 95% CI 1.019-1.604, *P* = 0.034), left main disease (HR 2.938, 95% CI 1.246-6.925, *P* = 0.014), small-caliber stents used (HR 2.207, 95% CI 1.189-4.095, *P* = 0.012), and total stent length (HR 1.057, 95% CI 1.005-1.112, *P* = 0.030). Serum sLOX-1 concentration ≥ 1.10 ng/ml had maximum sensitivity and specificity in predicting the occurrence of 2-year MACCEs (*P* < 0.001). Patients with higher serum sLOX-1 concentrations showed a significantly higher incidence of MACCEs in the Kaplan-Meier curve (*P* < 0.001). The combination of any of the risk factors identified in multiple Cox regression was associated with a stepwise increase in MACCE rate (*P* < 0.001).

**Conclusions:**

High baseline serum sLOX-1 concentration predicts 2-year MACCEs and shows an additional prognostic value to conventional risk factors in patients after primary PCI. sLOX-1 determination might play a complementary role in the risk stratification of patients with CAD treated with PCI.

## 1. Introduction

Cardiovascular diseases remain the leading cause of mortality and disability worldwide, with coronary artery disease (CAD) accounting for the greatest proportion [1]. Although the use of percutaneous coronary intervention (PCI) and new drug therapies has considerably improved the prognosis, patients with CAD remain at increased risk of major adverse cardiovascular and cerebrovascular events (MACCEs). Therefore, the early risk stratification of patients with high risk is important for the secondary prevention of MACCEs.

Lectin-like oxidized low-density lipoprotein receptor-1 (LOX-1) is a cell surface endocytosis receptor for atherogenic oxidized low-density lipoprotein receptor-1 (ox-LDL) [2]. LOX-1 is expressed in endothelial cells, macrophages, activated vascular smooth muscle cells, and atherosclerotic lesions [3]. It is involved in various critical steps of atherosclerosis, such as endothelial injury, leukocyte recruitment, foam cell formation, and plaque rupture [4]. Therefore, LOX-1 has been recognized as a potential therapeutic target for atherosclerotic disease [5, 6].

LOX-1 can be cleaved at the membrane proximal extracellular domain by some protease activities and released into the bloodstream as a soluble form (sLOX-1) [6, 7]. Epidemiological studies have demonstrated that sLOX-1 might be a predictive biochemical marker for CAD and stroke [8–10]. However, the long-term clinical effect of high serum sLOX-1 concentration on patients with CAD after primary PCI has not yet been fully investigated. Therefore, we aimed to investigate the association and prognostic value of elevated sLOX-1 concentrations with regard to long-term MACCEs in patients with CAD after primary PCI.

## 2. Materials and Methods

### 2.1. Study Population

From October 2015 to October 2016, 1074 consecutive patients who were treated with primary PCI and consented to be followed for 2 years in the Cardiology Department of 3 superior hospitals in Fujian Province (Union Hospital Affiliated to Fujian Medical University, The Second People's Hospital of Fujian Province, and the 476 Clinical Department of Fuzhou General Hospital) were recruited. The exclusion criteria were as follows: primary cardiomyopathy, diagnosis of acute autoimmune or inflammatory disease, unstable hemodynamics, advanced renal or hepatic disease requiring treatment, malignant disease, and symptomatic peripheral vascular diseases. After applying the inclusion and exclusion criteria, a total of 1011 patients were enrolled in this study. The protocol was approved by the research ethics committee at each participating center and is in accordance with the Declaration of Helsinki.

### 2.2. Primary PCI

All procedures were performed in the catheterization laboratory according to standard protocols. Before PCI, patients were pretreated with aspirin 300 mg and a loading dose of P2Y12 receptor antagonist. Unfractionated heparin was administered throughout PCI to maintain an activated clotting time of ≥250 seconds. Second-generation drug-eluting stents (DES) were implanted in all patients. Multivessel disease was defined as ≥2 main primary coronary artery stenosis greater than 50%. A small-caliber stent was defined as stent caliber ≤ 2.5 mm.

### 2.3. Blood Collection and Biochemical Analyses

Venous blood samples were taken from an antecubital vein of all patients in a fasting state before PCI. A set of blood samples were used for routine blood examination, to measure fasting blood glucose (FBG), triglyceride (TG), total cholesterol (TC), low-density lipoprotein cholesterol (LDL-c), high-density lipoprotein cholesterol (HDL-c), blood urea nitrogen (BUN), creatinine (Cre), creatine kinase-MB (CK-MB), uric acid (UA), NT-proBNP (N-terminal probrain natriuretic peptide), high-sensitivity CRP (hs-CRP), and homocysteine (Hcy) concentrations in the biochemical laboratory of hospitals. The other samples were centrifuged under the condition of 3000 r/min for 10 min. The serum was separated and then stored at -80°C for the detection of sLOX-1 concentrations. Serum sLOX-1 concentrations were measured by a commercially available enzyme-linked immunosorbent assay (ELISA) kit with an intra-assay CV of <10% and an interassay CV of <12% according to the manufacturer's protocols (USCN, Wuhan, China). All the samples were routinely analyzed by ELISA in duplicate, and the results were averaged to minimize measurement errors.

### 2.4. Follow-Up

After primary PCI, all patients were monitored for at least 24 hours. Patients were given standard medications including dual antiplatelet agents, statins, angiotensin-converting enzyme inhibitors or angiotensin II receptor, and beta-blocker by responsible physicians according to the up-to-date guidelines. After discharge, all patients were followed up for 2 years in an outpatient setting. The occurrence of MACCEs of all patients was identified by electronic patient records.

### 2.5. Study Endpoint

The primary endpoint of this study was the composite of MACCEs, which were identified as all-cause death, readmission for acute coronary syndrome (ACS), unplanned repeat revascularization, definite stent thrombosis, and ischemic stroke. ACS includes acute myocardial infarction (AMI) and unstable angina (UA) [11, 12].

### 2.6. Statistical Analyses

The study sample size was calculated by power analysis using preliminary data obtained in our laboratory with the following assumptions: an expected MACCE rate of 10% in the participants and a 0.4 ng/ml difference in mean sLOX-1 concentration between patients with and without MACCEs; therefore, at least 73 outcome events were needed with a power of 0.9 and a significance level (two-tailed) of 0.05 [13]. Data distribution patterns were analyzed using the Kolmogorov-Smirnov test. Normally distributed continuous variables were presented as mean ± SD, and continuous variables with a skewed distribution were expressed as median and interquartile range (25th to 75th percentile). Categorical and ordinal variables were presented as numbers and percentages. Comparison between groups was performed using the unpaired *t*-test, Mann-Whitney *U* test, chi-square test, or Fisher's exact text as indicated. Effects of factors on clinical outcomes after PCI were determined using multivariate Cox proportional hazard regression analysis. Receiver operating characteristic (ROC) curve and area under the curve (AUC) were explored to evaluate the diagnostic efficacy of factors. Overall MACCE rate was estimated using Kaplan-Meier survival curves with the log-rank test. *P* values less than 0.05 (two-tailed) were considered to indicate statistical significance. The Spearman rank correlation coefficient was used to determine the correlation between serum sLOX-1 concentrations and LDL-c/HDL-c concentrations. All data were analyzed by SPSS 22.0 for Windows (SPSS Inc., Chicago, Illinois, USA).

## 3. Results

### 3.1. Baseline Characteristics and Intergroup Comparisons

**Figure 1:**
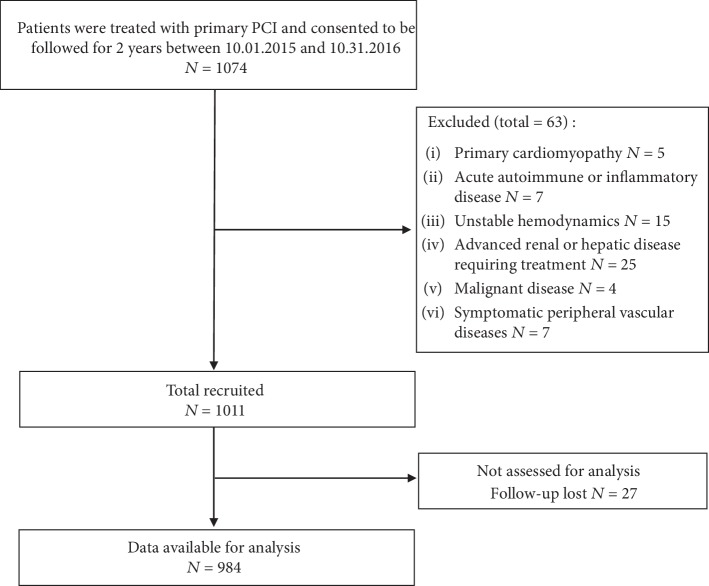
Study flowchart for participant selection.

The flowchart for patient selection is shown in Figure 1. The final study cohort included 984 patients undergoing primary PCI, including 768 patients with ACS (78.05%). Of all the 984 patients, 115 patients (11.69%) suffered from the combined endpoints, whereas 869 patients had no events (Table 1). The clinical, laboratory, and procedural characteristics between the MACCE and MACCE-free groups are shown in Table 2. Compared with the MACCE-free group, patients in the MACCE group had significantly lower DBP and higher sLOX-1 concentrations. Additionally, patients with MACCEs had higher total stent lengths and a higher prevalence of ACS, left main disease, and small-caliber stents used.

### 3.2. Multiple Cox Regression Analysis

Multiple Cox regression analysis was explored to identify the independent risk factors for MACCEs, and the results are shown in Table 3. The results revealed that 2-year MACCEs after PCI were associated with serum sLOX-1 concentrations (HR 1.278, 95% CI 1.019-1.604, *P* = 0.034), left main disease (HR 2.938, 95% CI 1.246-6.925, *P* = 0.014), small-caliber stents used (HR 2.207, 95% CI 1.189-4.095, *P* = 0.012), and total stent length (HR 1.057, 95% CI 1.005-1.112, *P* = 0.030).

### 3.3. ROC Curve Analysis

**Figure 2:**
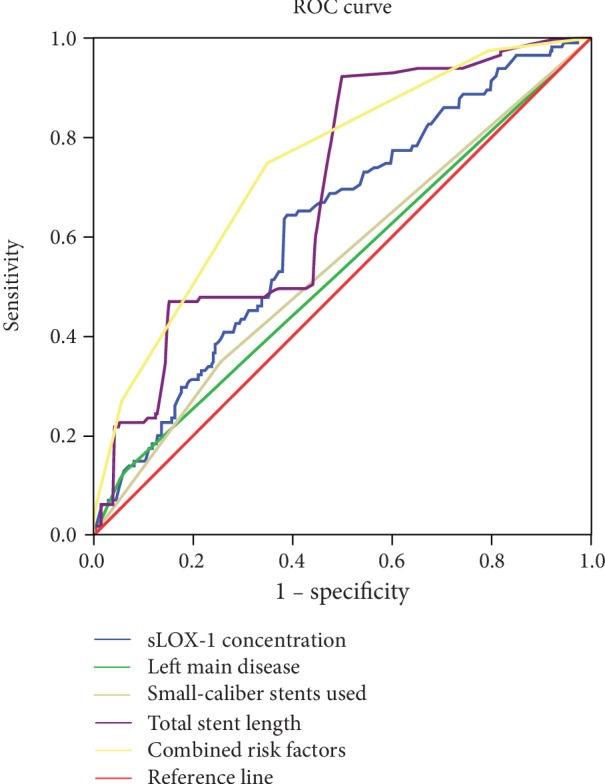
ROC curve analysis on predictive values of total stent length, sLOX-1 concentration, and combined risk factors. ROC curve = receiver operating characteristic curve, sLOX-1 = soluble lectin-like oxidized low-density lipoprotein receptor-1.

ROC curve analysis was used to evaluate the optimal cutoff values of the identified risk factors in predicting MACCEs. As shown in Figure 2, serum sLOX-1 concentration ≥ 1.10 ng/ml (AUC = 0.622, *P* < 0.001) and total stent length ≥ 32 mm (AUC = 0.692, *P* < 0.001) had maximum sensitivity and specificity in predicting the occurrence of 2-year MACCEs. Furthermore, combining all the risk factors resulted in a considerable improvement in AUC (AUC = 0.744, *P* < 0.001; Figure 2).

### 3.4. Kaplan-Meier Curve Analysis

**Figure 3:**
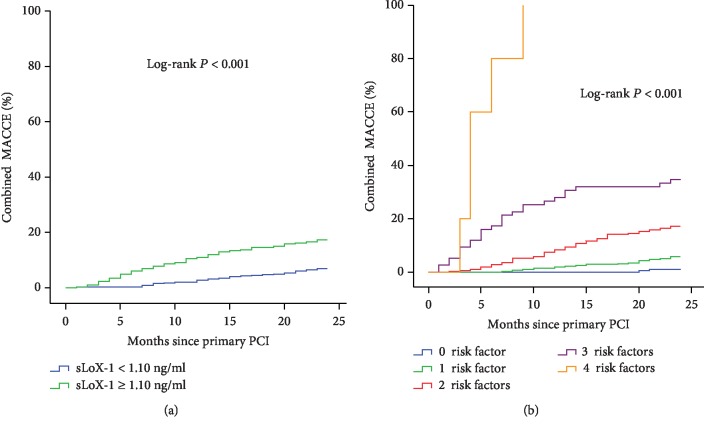
(a) sLOX-1 concentrations and 2-year MACCE rate after PCI. (b) Identified risk factors and 2-year MACCE rate after PCI. sLOX-1 = soluble lectin-like oxidized low-density lipoprotein receptor-1, MACCEs = major adverse cardiovascular and cerebrovascular events, PCI = percutaneous coronary intervention.

Serum sLOX-1 concentration was categorized into high (≥1.10 ng/ml) and low (<1.10 ng/ml) groups according to the cutoff value established in ROC curve analysis. As depicted in the Kaplan-Meier curve, patients with higher serum sLOX-1 concentrations showed a significantly higher incidence of MACCEs (log-rank *P* < 0.001, Figure 3(a)). Furthermore, the combination of any of the risk factors identified in multiple Cox regression was associated with a stepwise increase in the MACCE rate (log-rank *P* < 0.001, Figure 3(b)).

### 3.5. Correlations of sLOX-1 with LDL-c and HDL-c

**Figure 4:**
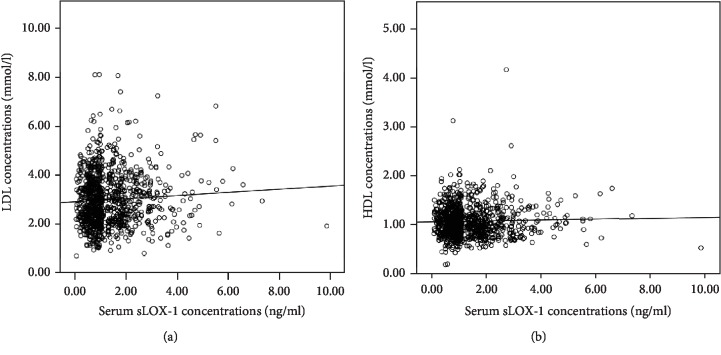
Correlations of sLOX-1 concentrations with LDL-c concentrations (a) and HDL-c concentrations (b). sLOX-1 = soluble lectin-like oxidized low-density lipoprotein receptor-1, LDL-c = low-density lipoprotein cholesterol, HDL-c = high-density lipoprotein cholesterol.

As shown in Figure 4, serum sLOX-1 concentrations were not correlated with LDL-c concentrations (*r* = 0.052, *P* = 0.104) and HDL-c concentrations (*r* = −0.009, *P* = 0.771).

## 4. Discussion

In this multicenter cohort study, we evaluated the association between baseline sLOX-1 concentration and long-term cardiovascular outcomes in patients undergoing PCI. We demonstrated that (1) high baseline serum sLOX-1 concentration is an independent predictor of 2-year MACCEs after primary PCI, (2) patients with higher serum sLOX-1 concentrations showed significantly higher incidence of MACCEs than patients with lower serum sLOX-1 concentrations, and (3) sLOX-1 showed an additional prognostic value to conventional risk factors.

In the present study, the overall incidence rate of MACCEs in patients after primary PCI was in broadly consistent with that reported in previous studies [14, 15]. The relatively higher MACCE rate served as the impetus to identify specific biomarkers that can be easily measured and provide prognostic information beyond that of conventional risk factors. Moreover, the identification of reliable biomarkers can assist in determining possible new therapeutic targets and thus facilitate therapeutic decision.

Here, we demonstrated that sLOX-1 concentrations were significantly higher in patients suffering from MACCEs compared to event-free patients. However, serum sLOX-1 concentrations were correlated with neither LDL-c nor HDL-c concentrations. This result may be attributed to the fact that, in addition to ox-LDL, LOX-1 can also be upregulated by several risk factors for CAD including hemodynamic stress, angiotensin II, and inflammatory stimuli [4–6]. Circulating sLOX-1 may reflect the total burden of coronary atherosclerosis or identify high-risk atherosclerotic lesions that are prone to rupture [10]. Several studies have demonstrated that serum sLOX-1 concentrations were significantly elevated in ACS patients and associated with adverse clinical outcomes [8, 16]. We have previously found that serum sLOX-1 concentrations were correlated with angiographically complex lesions in patients with CAD [17]. Various studies have shown that a complex lesion was the predictor of adverse outcome in patients with CAD [18, 19]. Higher concentrations of sLOX-1 were also observed in patients with acute stoke compared to controls [20]. Therefore, the result that MACCE groups had increased serum sLOX-1 concentrations was in accordance with previous studies.

Survival analysis was employed to evaluate the association between sLOX-1 concentrations and clinical outcomes. Multiple Cox regression analysis revealed that sLOX-1 concentration was the independent risk factor for MACCEs. Kaplan-Meier survival curves, based on the sLOX-1 cutoff of 1.10 ng/ml established in ROC curve analysis, showed early and persistent separation during 2-year follow-up. These findings indicated that elevated baseline sLOX-1 concentration is associated with adverse clinical outcomes after PCI. sLOX-1 concentration may be useful for the risk stratification of patients with CAD undergoing PCI.

Various potential mechanisms may explain the association between elevated sLOX-1 concentration and long-term adverse clinical outcomes observed in the present study. LOX-1 can be upregulated rapidly via the mechanical stimulation after stent implantation [21]. Its activation is implicated in the pathophysiological processes that are responsible for restenosis, including vascular smooth muscle cell (VSMC) migration and proliferation [22]. Two separate studies have demonstrated that circulating sLOX-1 concentrations were associated with in-stent restenosis in patients after PCI [23, 24]. LOX-1 activation may initiate atherosclerotic lesion formation and contribute to plaque rupture [25]. Balin et al. have found that increased circulating sLOX-1 concentrations were correlated with periprocedural myocardial infarction (PMI) in patients with stable CAD undergoing elective PCI [26]. PMI is an important contributor to the morbidity and mortality associated with PCI [27].

Left main disease, the use of small-caliber stents, and total stent length were well-established risk factors for MACCEs in patients treated with PCI [28–31]. All these risk factors were validated in the present study via multiple Cox regression analysis. The Kaplan-Meier survival curve analysis showed how elevated sLOX-1 concentration augmented the association of conventional risk factors with 2-year MACCEs. The ROC curve analysis demonstrated that the combination of sLOX-1 concentration with the conventional risk factors was more predictive than individual markers in predicting 2-year MACCEs. If validated, then these results may have important clinical implications because high-risk patients can benefit from a more intense and individualized treatment plan.

This study should be interpreted by considering several potential limitations. First, the nonrandomized design and a modest sample size of the present study cannot rule out the residual confounders and selection bias. The numbers of patients with and without MACCE exhibit considerable variance, and thus, the accuracy of this study in concluding an exact prognostic association is low. Therefore, the findings of the present study will require further validation and qualification in a large, longitudinal cohort. Second, only serum sLOX-1 concentrations were analyzed in this study. The investigation of other potential biomarkers can provide additional information on the prognostic value of sLOX-1. Third, several scoring systems, such as GRACE and SYNTAX scores, were established for the risk stratification of patients undergoing PCI. The additive prognostic value of sLOX-1 concentration to such scoring systems is an interesting topic for investigation.

In conclusion, high serum sLOX-1 concentration predicts 2-year MACCEs and provides an additional prognostic value to conventional risk factors in patients after primary PCI. If future studies will prove the causality of the observation, sLOX-1 determination might play a complementary role in the risk stratification of patients with CAD treated with PCI.

## Figures and Tables

**Table 1:** MACCEs during 2-year follow-up.

Events	*n*	% of patients with MACCEs	% of all patients
All-cause death	7	6.09	0.71
Readmission for ACS	41	35.65	4.17
Unplanned repeat revascularization	53	46.09	5.39
Definite stent thrombosis	1	0.87	0.10
Ischemic stroke	13	11.30	1.32
In total	115	100	11.69

MACCE = major adverse cardiovascular and cerebrovascular event, ACS = acute coronary syndrome.

**Table 2:** Clinical, laboratory, and procedural characteristics in patients with and without MACCEs.

	MACCE-free (*n* = 869)	MACCE (*n* = 115)	*P* value
Clinical characteristics			
Age (years)	67 (59-74)	69 (59-76)	0.215
Male, *n* (%)	679 (78.14)	81 (70.43)	0.064
BMI	24.17 ± 2.87	24.65 ± 2.92	0.099
SBP (mmHg)	130 (120-144)	128 (119-142)	0.239
DBP (mmHg)	80 (70-86)	75 (68-83)^∗^	0.016
LVEF (%)	63 (56-68)	63 (55-69)	0.697
Laboratory characteristics			
FBG (mmol/l)	5.64 (5.01-6.82)	5.54 (4.95-6.58)	0.437
TG (mmol/l)	1.35 (0.98-1.94)	1.44 (1.10-2.00)	0.171
TC (mmol/l)	4.34 (3.62-5.21)	4.38 (3.48-5.46)	0.772
LDL-c (mmol/l)	2.85 (2.23-3.62)	2.96 (2.22-3.85)	0.517
HDL-c (mmol/l)	1.01 (0.85-1.21)	1.04 (0.86-1.19)	0.727
BUN (mmol/l)	4.90 (4.10-6.00)	5.20 (4.10-6.10)	0.162
Cre (*μ*mol/l)	77 (67-91)	76 (69-87)	0.891
CK-MB (U/l)	15.30 (11.60-22.90)	16.10 (12.00-69.20)	0.096
UA (*μ*mol/l)	352 (295-417)	355 (300-420)	0.823
NT-proBNP (ng/l)	237 (69-831)	206 (51-921)	0.319
Hs-CRP (mg/l)	3.17 (0.96-8.73)	2.61 (0.98-11.10)	0.715
Hcy (*μ*mol/l)	9.36 (7.70-11.75)	9.18 (7.75-11.42)	0.870
sLOX-1 (ng/ml)	1.00 (0.67-1.77)	1.38 (0.90-2.16)^†^	<0.001
Cardiovascular risk factors			
Smoking, *n* (%)	417 (47.99)	61 (50.04)	0.308
DM, *n* (%)	280 (32.22)	36 (31.30)	0.843
Hypertension, *n* (%)	548 (63.06)	67 (58.26)	0.318
ACS, *n* (%)	669 (76.99)	99 (86.09)^∗^	0.027
Cardiovascular medication			
Aspirin, *n* (%)	853 (98.16)	114 (99.13)	0.452
Clopidogrel, *n* (%)	782 (89.99)	102 (88.70)	0.666
Ticagrelor, *n* (%)	86 (9.90)	13 (11.30)	0.637
Statins, *n* (%)	852 (98.04)	112 (97.39)	0.641
ACEI/ARB, *n* (%)	621 (71.46)	88 (76.52)	0.256
Beta-blocker, *n* (%)	690 (79.40)	90 (78.26)	0.777
Procedural characteristics			
Number of affected segments	2 (1-3)	2 (1-4)	0.071
Left main disease, *n* (%)	50 (5.75)	14 (12.17)^†^	0.009
Multivessel disease, *n* (%)	466 (53.62)	70 (60.87)	0.143
Stents per procedure	2 (1-2)	2 (1-2)	0.117
Small-caliber stents used, *n* (%)	222 (25.65)	40 (33.61)^∗^	0.035
Total stent length (mm)	30 (23-54)	38 (33-66)^†^	<0.001
Pre-PCI stenosis (%)	90 (85-100)	90 (80-100)	0.467
Post-PCI stenosis (%)	0 (0-0)	0 (0-0)	0.414
Initial TIMI flow	3 (2-3)	3 (1-3)	0.449
Final TIMI flow	3 (3-3)	3 (3-3)	0.407
Chronic total occlusion lesion, *n* (%)	103 (11.85)	15 (13.04)	0.712
Bifurcation lesion, *n* (%)	89 (10.24)	9 (7.83)	0.416

All values are presented as median value (interquartile range) or *n* (%). BMI = body mass index, SBP = systolic blood pressure, DBP = diastolic blood pressure, LVEF = left ventricular ejection fraction, FBG = fasting glucose, TG = triglycerides, TC = total cholesterol, LDL-c = low-density lipoprotein cholesterol, HDL-c = high-density lipoprotein cholesterol, BUN = blood urea nitrogen, Cre = creatinine, CK-MB = creatine kinase-MB, UA = uric acid, NT-proBNP = N-terminal probrain natriuretic peptide, Hs-CRP = high-sensitivity C-reactive protein, Hcy = homocysteine, sLOX-1 = soluble lectin-like oxidized low-density lipoprotein receptor-1, DM = diabetes mellitus, ACEI = angiotensin-converting enzyme inhibitor, ARB = angiotensin receptor blocker, PCI = percutaneous coronary intervention, TIMI = thrombolysis in myocardial infarction. Other abbreviations are shown in Table 1. ^∗^*P* < 0.05 compared to patients without events, ^†^*P* < 0.01 compared to patients without events.

**Table 3:** Risk factors for 2-year MACCE in multiple Cox regression.

Factors	HR (95% CI)	*P* value
sLOX-1	1.278 (1.019-1.604)	0.034
Left main disease	2.938 (1.246-6.925)	0.014
Small-caliber stents used	2.207 (1.189-4.095)	0.012
Total stent length	1.057 (1.005-1.112)	0.030

HR = hazard ratio, CI = confidence interval. Other abbreviations are shown in Tables 1 and 2.

## Data Availability

We claimed that the datasets generated during and/or analyzed during the current study are available from the corresponding author on reasonable request.
